# Gender differences in the experience of interstitial cystitis/bladder pain syndrome

**DOI:** 10.3389/fpain.2022.954967

**Published:** 2022-08-11

**Authors:** Sula S. Windgassen, Susanna Sutherland, Michael T. M. Finn, Kemberlee R. Bonnet, David G. Schlundt, W. Stuart Reynolds, Roger R. Dmochowski, Lindsey C. McKernan

**Affiliations:** ^1^Department of Health Psychology, Institute of Psychiatry, Psychology and Neuroscience, King's College London, London, United Kingdom; ^2^Department of Psychiatry and Behavioral Sciences, Vanderbilt University School of Medicine, Nashville, TN, United States; ^3^Helen DeVos Children's Hospital, Grand Rapids, MI, United States; ^4^Department of Pediatrics and Human Development, Michigan State University, Grand Rapids, MI, United States; ^5^Department of Psychology, Vanderbilt University, Nashville, TN, United States; ^6^Department of Urologic Surgery, Vanderbilt University School of Medicine, Nashville, TN, United States; ^7^Department of Physical Medicine and Rehabilitation, Vanderbilt University School of Medicine, Nashville, TN, United States

**Keywords:** interstitial cystitis, lower urinary tract symptom (LUTS), sex characteristics, sexism, patient acceptance of health care, psychosocial intervention, qualitative—quantitative analysis

## Abstract

**Aims:**

This study assessed gender differences in a debilitating urologic pain condition, interstitial cystitis/bladder pain syndrome (IC/BPS). We aimed to (1) evaluate how pain, symptom, and distress profiles of IC/BPS may differ between genders and (2) obtain in-depth firsthand accounts from patients to provide additional insight into their experiences that may explain potential gender differences.

**Methods:**

A mixed methods approach combined validated patient-reported outcome measures with a single timepoint 90-min focus group. Tests of summary score group differences between men and women were assessed across questionnaires measuring urologic symptoms, pain, emotional functioning, and diagnostic timeline. Qualitative analysis applied an inductive-deductive approach to evaluate and compare experiences of living with IC/BPS Group narratives were coded and evaluated thematically by gender using the biopsychosocial model, providing insight into the different context of biopsychosocial domains characterizing the male and female experience of IC/BPS.

**Results:**

Thirty-seven participants [women (*n* = 27) and men (*n* = 10)] completed measures and structured focus group interviews across eight group cohorts conducted from 8/2017 to 3/2019. Women reported greater pain intensity (*p* = 0.043) and extent (*p* = 0.018), but not significantly greater impairment from pain (*p* = 0.160). Levels of psychological distress were significantly elevated across both genders. Further, the duration between time of pain symptom onset and time to diagnosis was significantly greater for women than men (*p* = 0.012). Qualitative findings demonstrated key distinctions in experiences between genders. Men appeared not to recognize or to deter emotional distress while women felt overwhelmed by it. Men emphasized needing more physiological treatment options whilst women emphasized needing more social and emotional support. Interactions with medical providers and the healthcare system differed substantially between genders. While men reported feeling supported and involved in treatment decisions, women reported feeling dismissed and disbelieved.

**Conclusion:**

The findings indicate different pain experiences and treatment needs between genders in persons experiencing urologic pain and urinary symptoms, with potential intervention implications. Results suggest gender health inequality in medical interactions in this urologic population needing further investigation.

## Introduction

Chronic pain, or pain that persists or recurs for 6 months or more, affects mental and physical health and generates both disability and suffering (1, 2). The literature indicates that there may be common mechanisms of chronic pain across conditions, with increasing research exploring the role of central sensitization (3, 4). It is now well-established that the best understanding of what chronic pain is and how it is maintained is from a biopsychosocial perspective (2, 5). It is therefore important to understand the multiple biological, psychological and social factors that may interact, ultimately affecting pain outcomes (4).

Gender is a characteristic inherently overlapping biology, sociology and psychology. Gender moderates the experience of pain, with greater prevalence, sensitivity to, and intensity of chronic pain reported in female populations (6, 7). Less is understood about why this occurs. A better understanding of how gender influences a person's experience of pain could enhance mechanism and intervention research (6) and influence personalized medicine efforts for its management (4).

Interstitial cystitis/bladder pain syndrome (IC/BPS) is a debilitating chronic pain condition characterized by pain, pressure or discomfort in the bladder and pelvic region coupled with urinary urgency or frequency (8). IC/BPS affects 3–8 million women and 1–4 million men in the United States (9, 10). Previously it was thought that IC/BPS was 10 times more likely to occur in women than men (11), but more recent research suggests that prevalence in men approaches that in women (10). IC/BPS may be underdiagnosed in men, and is often grouped with another similar condition called chronic prostatitis/chronic pelvic pain syndrome (CP/CPPS) (10, 12). Leading research networks recently adopted the term “urologic chronic pelvic pain syndrome” (UCPPS) to encompass both IC/BPS and CP/CPPS based on their similarities (13).

There is a significant psychological burden of IC/BPS, including high rates of depression, anxiety, and suicidal ideation that can worsen symptoms and prognosis (14, 15). National guidelines recommend psychological interventions as first line treatment for IC/BPS (16). This is a new area of study with very little intervention research available (15). It is therefore important to understand how such interventions may need to be tailored according to gender-based needs. Our recent study exploring women's experience of IC/BPS found that symptom severity was significantly associated with depression and that there was a reciprocal relationship between psychosocial stress and symptom exacerbations (17). Key qualitative themes included the experience of invalidation from loved ones and healthcare providers, social withdrawal, isolation, and a perceived lack of control over symptoms (17). To our knowledge there are no studies exploring men's experience of IC/BPS. Given that IC/BPS affects proportionately more women than men, with significant psychological impact, comparison of men's experience of this condition could elucidate important biopsychosocial considerations for intervention development.

We therefore sought to explore whether and how experiences of pain in IC/BPS and consequent treatment needs differ between men and women with a mixed methods approach. Using qualitative analysis provides an in-depth assessment of patient experiences necessary to understand the impact and perceived needs in IC/BPS due to the limited existing research. We sought to highlight salient differences between genders informed by our previous qualitative assessment of women's experience of IC/BPS (17), by replicating methods and comparing findings by gender.

## Methods

The institutional review board reviewed and approved all procedures of this investigation. Patients with IC/BPS were recruited at outpatient clinics in a large academic medical center using hospital databases and an online national clinical research participation repository (18).

Participants were screened for eligibility prior to enrolment by referring medical providers or trained research staff. Inclusion criteria were English speaking adults (age >18) with a diagnosis of IC/BPS later confirmed through medical record review of clinical provider notes. If medical records were unavailable, validated cutoff scores of urologic symptom assessments were applied in addition to participant self-report to indicate the presence of IC/BPS (17). Exclusion criteria included the presence of conditions that would interfere with focus group participation or reliable completion of assessment measures such as psychotic disorder, cognitive impairment, substance dependence, acute emotional distress, or active suicidal ideation. Further details of the study process are published elsewhere (17). Participants were invited to participate in a 90-min focus group conducted by an experienced qualitative research group facilitator (KB) following a structured guide with a set of pre-determined questions and prompts. Groups were separated by a person's identified gender. Prior to the group, all participants completed consenting procedures and the below series of validated questionnaires.

### Study procedures

#### Patient-reported outcomes measurement

The O'Leary-Sant Symptom (ICSI) and Problem Indexes (ICPI) (19) measured IC/BPS symptom severity. The short form Brief Pain Inventory (BPI) (20) measured pain interference and intensity. The Michigan Body Map (MBM) (21) measured extent of pain. The Patient Health Questionnaire −9 (PHQ-9) (22) measured depression, and the PROMIS Anxiety short form (23) measured anxiety. These measures have acceptable reliability and validity in their target domains. See Mckernan et al. (17) for more detailed information regarding each of these measures.

#### Quantitative analysis

Descriptive statistics assessed demographic data. Tests of normality (Q-Q plots, Kolmogorov-Smirnov Statistic) confirmed normality of distribution in the small sample size and tested for the need for transformation or analytic bootstrapping. Due to sample size and the distribution of scores, authors employed tests of summary statistics to examine group differences, which has been found to result in clinically-meaningful results for interpretation (24). Scores of each measure were plotted by gender for visual comparison of score distributions.

#### Qualitative analysis

Qualitative data coding and analysis was managed by the Institutional Qualitative Research Core, led by a PhD-level psychologist (DS). Data coding and analysis followed standard consolidated criteria for Reporting Qualitative Research Guidelines (25). A hierarchical coding system was developed and refined using the focus group guide and a preliminary review of the transcripts. Major coding categories included: (1) living with IC/BPS; (2) social/mental health support; (3) treatment experiences; (4) provider capabilities; and (5) treatment needs. These main categories were further divided into subcategories for both genders, with some subcategories having additional levels of hierarchical divisions. Definitions and rules were written for the use of each category.

#### Coding and examination of transcripts

**Figure 1:**
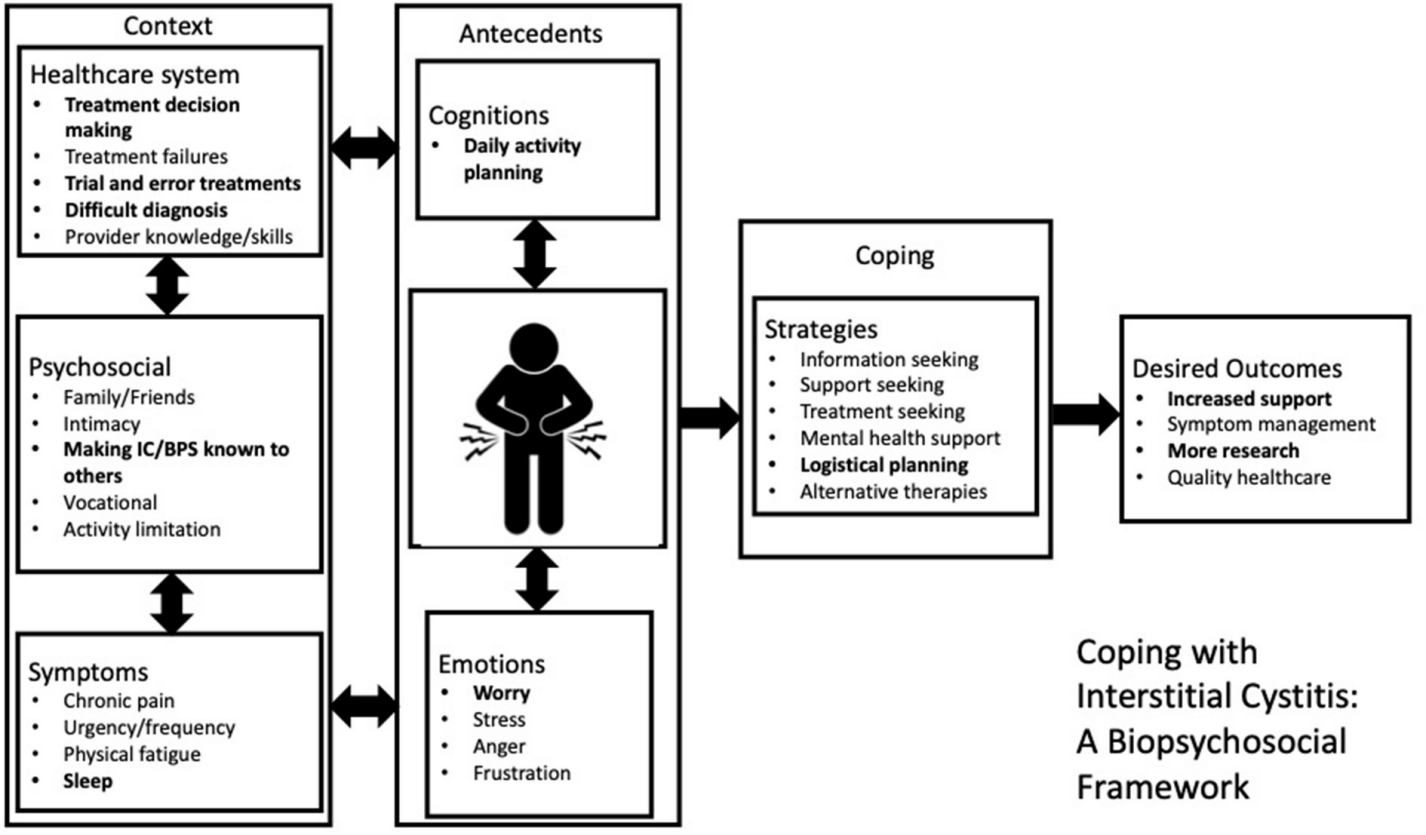
Biopsychosocial conceptualization of men's experience with interstitial cystitis.

Inter-coder reliability was established by two experienced qualitative data coders. Two separate coders reviewed all de-identified transcribed data, separating and coding each participant statement by theme and subtheme. Each participant statement could be assigned up to five codes. The coded themes for both genders were organized by number of mentions across focus groups. Any coding discrepancies were resolved through collaborative discussion as is common practice in qualitative research (26). Data coding and analysis was conducted using SPSS 26 software and Microsoft Excel 2016. An inductive-deductive approach (26) was used to develop a conceptual framework utilizing the widely accepted biopsychosocial model of pain 5 (Figure 1), which the focus group data could inductively provide more specific insight into for both men and women.

## Results

**Figure 2:**
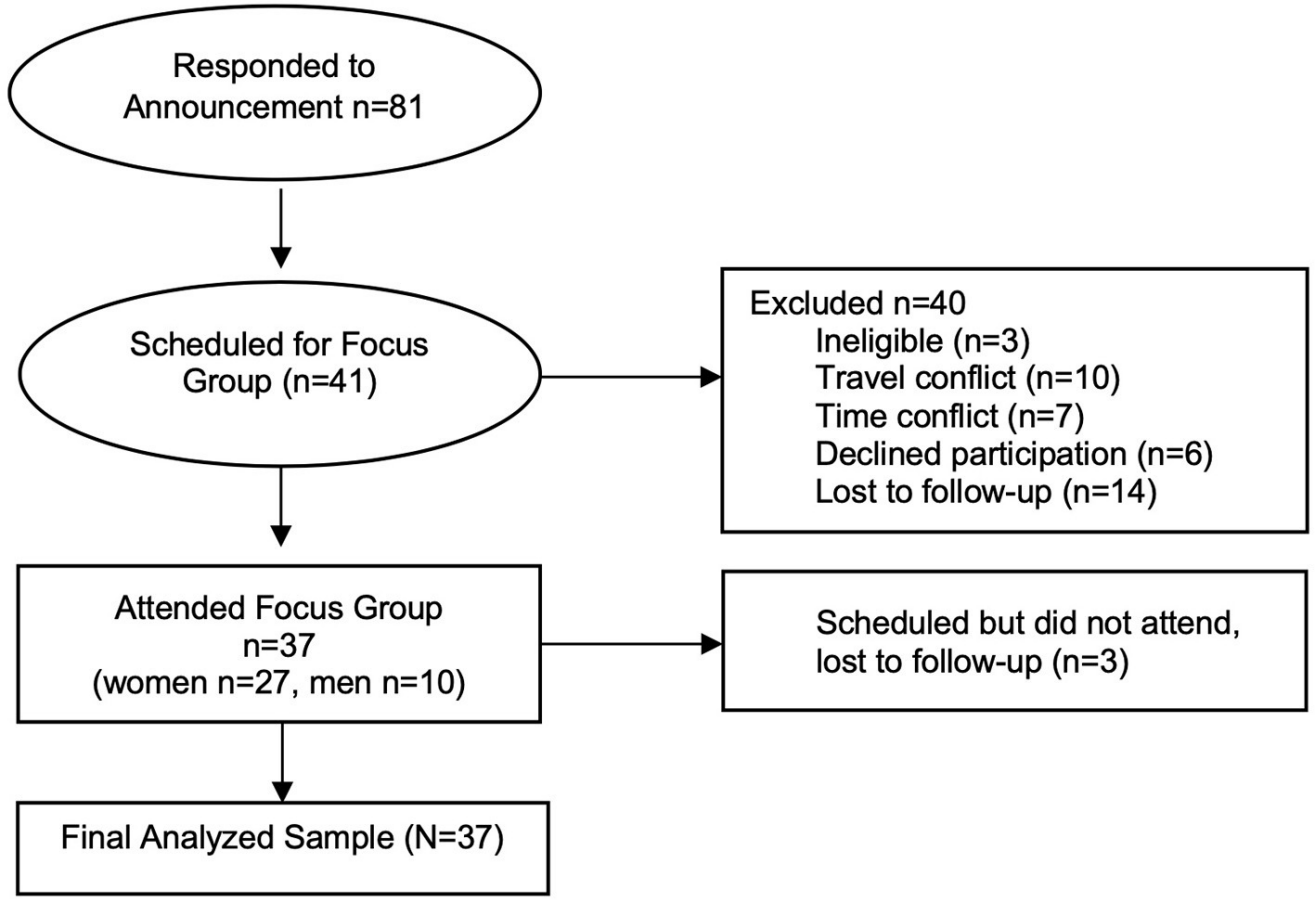
Study flow.

Figure 2 indicates study flow. Eighty-two individuals responded to study advertisements. Of these individuals, 42 agreed to participate in the study, with 37 ultimately consenting and undergoing all study procedures (45% enrollment rate). Eight focus groups occurred, six with women and two with men. Participant age averaged 46.59 years. The sample was 73.0% female, largely college-educated (60.2%), and predominately white (89.0%). Demographic factors were similar between both genders (Table 1), with the exception of marital status with men being more likely to be single.

**Table 1:** Demographic and clinical characteristics of participants.

**Variable**	**Total (*N* = 37)**	**Women (*n* = 27)**	**Men (*n* = 10)**	**Tests of gender differences**		
	**Mean (SD)/Count (%)**	**Mean (SD)/Count (%)**	**Mean (SD)/Count (%)**	**F/t**	**df**	***p***
**Demographic**
Age	46.59 (16.31)	45.00 (16.30)	50.90 (16.38)	0.974	35	0.334
Ethnicity				−0.828	1	0.413
White	33 (89%)	23 (85.25%)	10 (100%)			
Black	2 (5.4%)	2 (7.4%)	0 (0%)			
Multiracial or non-listed	2 (5.4%)	2 (8.4%)	0 (0%)			
Marital status				−0.394	1	0.696
Single (Never Married)	14 (37.8%)	10 (37.04%)	4 (40%)			
Married or domestic partnership	15 (40.5%)	10 (37.04%)	5 (50%)			
Divorced/widowed	8 (21.6%)	7 (25.93%)	1 (10%)			
Education				1.094	1	0.281
High school diploma or equivalent	4 (10.8%)	3 (11.11%)	1 (10%)			
Vocational/technical school	3 (8.1%)	3 (11.11%)	0 (0%)			
Some college	4 (10.8%)	4 (14.81%)	0 (0%)			
Bachelor's degree	16 (43.2)	10 (37.04%)	6 (60%)			
Master's degree	6 (16.2%)	5 (18.52%)	1 (1%)			
Doctorate or professional degree	4 (10.8%)	2 (7.41%)	2 (20%)			
**Clinical**
Intersticial cystitis symptom inventory	15.57 (5.07)	16.44 (4.79)	13.20 (5.27)	−1.800	34	−0.081
Intersticial cystitis problem inventory	13.35 (4.20)	14.19 (3.98)	11.10 (4.12)	−2.127	34	−0.041
Michigan body map	8.78 (10.06)	10.52 (11.01)	11.10 (7.05)	−2.47	34	0.018
BPI intensity	15.65 (9.29)	17.33 (9.55)	4.10 (4.56)	−2.145	34	−0.043
BPI interference	26.24 (19.22)	28.92 (19.67)	19.00 (16.71)	−1.436	34	0.16
PROMIS anxiety	19.67 (9.17)	20.76 (8.29)	16.8 (7.48)	−1.361	34	0.182
PHQ-9	7.56 (7.33)	8.46 (7.27)	5.2 (7.33)	1.288	33	0.206
Age of diagnosis	39.08 (17.07)	37.70 (17.27)	42.80 (16.81)	0.79	34	0.435
Age of first symptoms	33.05 (17.23)	30.26 (17.15)	40.60 (15.83)	1.702	34	0.098
Time lag between onset and diagnosis	6.03 (8.72)	7.44 (9.78)	2.20 (2.25)	−2.678	31	0.012

Percentages and analyses of household income exclude those who responded “Rather not say.” For assessing differences between genders on household income and education, wilcoxon rank sum test used to explore group differences, treating the dependent variable as ordinal.

### Quantitative

#### Quantitative examination of gender differences

Demographic means and standard deviations are reported in Table 1. Independent samples *t*-tests of summary statistics did not reveal any significant differences between genders regarding race, *t*_(34)_ = 0.828, *p* = 0.413, marital status, *t*_(34)_ = −0.394, *p* = 0.696, or education, *t*_(34)_ = 1.094, *p* = 0.281. Data distributions appeared normal based on inspection of Q-Q plots and Kolmogorov-Smirnov tests, with the exception of MBM. Therefore, the MBM scores were log transformed before further analysis.

##### Urinary symptom profiles

**Figure 3:**
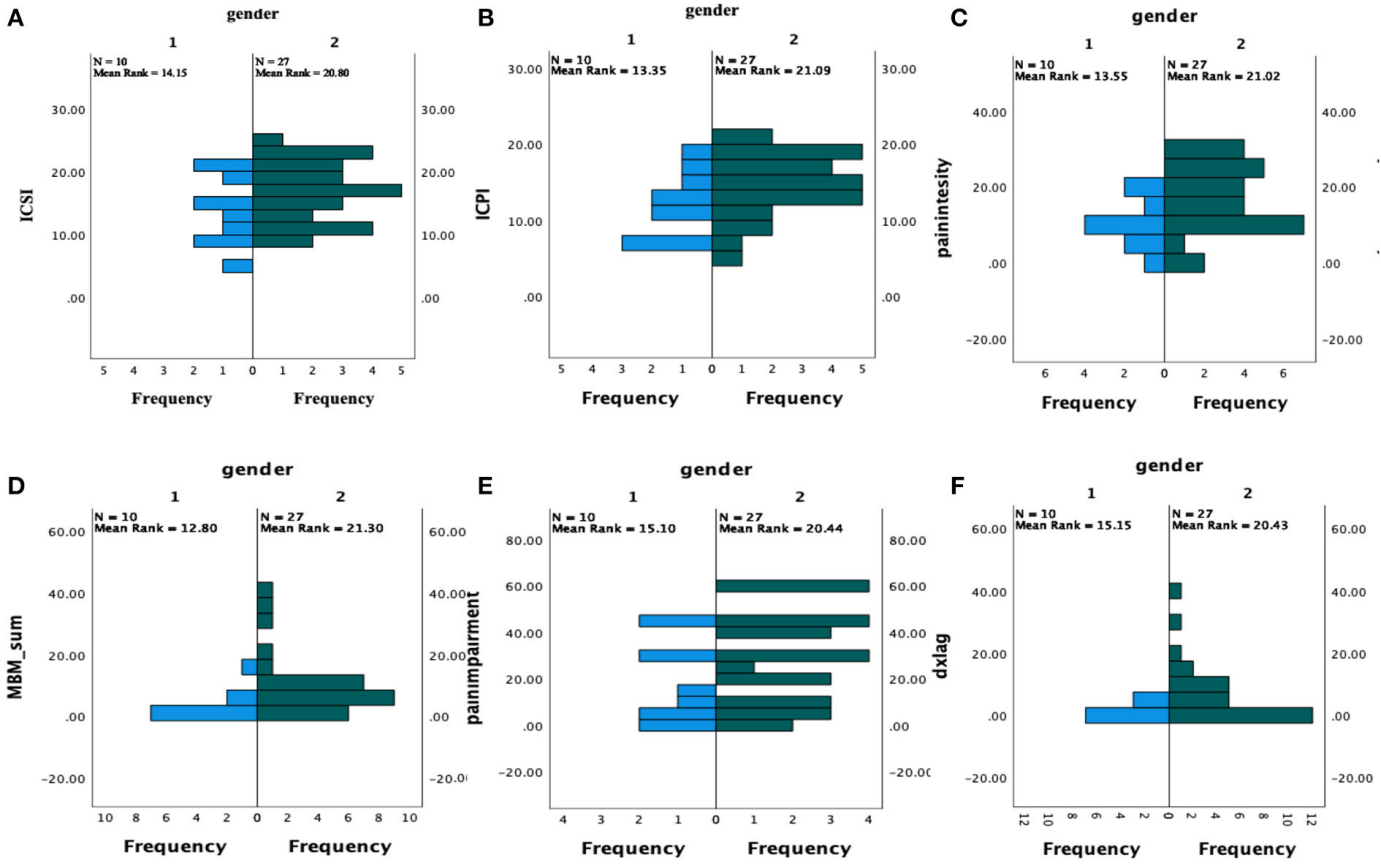
Symptom comparison by Gender. 1 = men (*n* = 10), 2 = women (*n* = 27), ICSI, interstitial cystitis symptom index; ICPI, interstitial cystitis problem index; MBM, michigan body map; dxlag, time-to-diagnosis from symptom onset. **(A)** ICSI by gender; **(B)** ICPI by gender; **(C)** Intensity by gender; **(D)** Michigan body map pain area count; **(E)** Brief pain inventory impairment by gender; **(F)** Time lag between symptom onset and diagnosis by gender.

Independent samples *t*-tests of summary statistics revealed a trend toward significant differences between genders regarding severity of urologic symptoms, *t*_(34)_ = −1.800, *p* = 0.081, with women reporting non-significantly elevated mean symptoms. Significant differences between genders regarding problems due to urologic symptoms indicated women reported significantly greater problems due to their symptoms than men, *t*_(34)_ = −2.127, *p* = 0.041 (Figure 3).

##### Pain symptom profiles

Independent samples *t*-tests of summary statistics revealed varying profiles across pain indices, with women reporting significantly greater pain intensity [*t*_(34)_ = −2.146, *p* = 0.043] and extent [*t*_(34)_ = −2.47, *p* = 0.018] than men. However, pain interference did not differ significantly by gender, *t*_(34)_ = −1.436 *p* = 0.160 (Figure 3).

##### Psychological symptom profiles

Raw symptoms of depression were significantly elevated in both men and women to a “mild” degree (*M* = 5.2, 8.5). After following standardized PROMIS protocol to convert raw data to T-scores, symptom elevations indicated significant anxiety for both men (*T* = 56, “mild anxiety”) and women (*T* = 61, “moderate anxiety”). However, independent samples *t*-tests of summary statistics did not reveal any significant differences between genders regarding symptoms of depression, *t*_(33)_ = 1.288, *p* = 0.206, or anxiety *t*_(34)_ = −1.361, *p* = 0.182.

##### Diagnostic differences

Independent samples *t*-tests of summary statistics did not reveal any significant differences between genders regarding age of diagnosis, *t*_(34)_ = 0.790, *p* = 0.435, or age of symptom onset, *t*_(34)_ = 1.702, *p* = 0.098. However, the time difference between symptom onset and disorder diagnosis was significantly greater for women than for men, *t*_(31)_ = −2.678, *p* = 0.012 (Figure 3).

### Qualitative

Figure 1 illustrates the biological, psychological, and environmental circumstances that influence IC/BPS coping strategies for men. The left-hand side represents the important contextual factors pertaining to the healthcare system, psychosocial, and IC/BPS symptoms. The center box shows the immediate influence of cognition and emotion on selection of coping strategies. The far-right box shows important outcomes that are modified through use of coping strategies. The double arrows depict the dynamic interplay among cognition, affect, and behaviors. The experiences that men reported but women did not are in bold.

Five main domains are explored within the above framework: (1) Symptom experience and impact (2) healthcare system (3) emotional symptoms (4) coping (5) addressing treatment needs. Each element of the conceptual framework is presented in further detail below.

#### Symptom experience and impact

Table 2 contains illustrative quotes provided by the men within each theme. Men described the chronicity and severity of IC symptoms, which included urinary pain and frequency and fatigue due to sleep disruption (*quotation 1.01*). Although urinary pain was described (quotation 1.02), urinary frequency was more of a focal point in terms of the impact on life. This contrasted with women who commonly cited bladder pain as the most difficult symptom to deal with (“*the burning was the worst for me*”) (17). Interactions with comorbid conditions were also discussed as challenging due to management strategies for comorbid conditions having unintended consequences for IC/BPS (*quotation 1.03*). Comorbidity was also a perceived barrier to receiving a diagnosis of IC/BPS sooner by some (*quotation 1.04*), as symptoms were attributed to the comorbidity (diabetes). One participant considered treatment of his comorbid condition as having a positive effect on reducing urinary frequency at night (*quotation 1.05*).

**Table 2:** Table of quotes for each theme.

**Theme 1**	**Symptom experience & impact**	
**Quote number**	**Quote**	**Participant**
1.01	“Frequency of the urination has just totally worn me out. The maximum I've been was probably 40–50 times a day. It's now down to about 30. But, the night times is what causes me problems. I'm up anywhere from 6 to 10 times a night. I have been up, when I was tracking it, the highest I had ever been up, I was up 24 times one night”.	Participant 1, Group 1
1.02	“It got down to a point where I was burning all the time, so I couldn't hardly live with that.”	Participant 1, Group 1
1.03	“If I have a lot of salt, I won't have to go as much. Well, I don't want to do that because I've got blood pressure problems. I have to be careful with the salt, I know though, if I eat salt then I may not have to go for 2 h, wouldn't that be wonderful. And so, it is about the frequency. It's really controlling that and managing that and dealing with that more than anything else, I would say.”	Participant 4, Group 1
1.04	“When I first went to the doctor, he said that he thought it was related to my diabetes. I'm a type 2 diabetic, but I maintain my A1C at very low levels. So I said, ”Okay, we'll try to eat better.	Participant 1, Group 1
1.05	“Interestingly enough, about 2 years ago, I had to have a sleep apnea test, I started using a sleep apnea machine, a CPAP machine. Interestingly enough, after I started using a sleep apnea machine, my getting up and going to the bathroom at night was cut from three or four times down to one, is a good night.”	Participant 4, Group 1
1.06	“I always went to work and it cost me and they got rid of me because I wasn't performing the way I should've. I was making bad choices. Bad decisions.”	Participant 3, Group 1
1.07	“Traffic has got so horrible. A friend of mine's like, “Come over.” It's like, “I can't, I have to wait a couple hours because there's no way I can drive across (city) right now to go visit. I can't do it.”	Participant 3
1.08	“And our relations have not been good at all from a standpoint, just sexual relations. So, I've pretty much given up on that end of it for now. Simply because I'm more concerned about getting to the bottom of this problem”	Participant 1, Group 1
1.09	“What makes me mad is that, like last year on the trip, we were just getting ready to board and my kids said, “Dad, just relax. It's in your mind.” And, “Just calm down. You're making it worse.”'	Participant 1, Group 1
1.10	“[I] sort of withdrew a little bit and didn't see some people probably as much as I would have liked to. Just because I had different things that I had to consider…”	Participant 2, Group 1
**Theme 2**	**Healthcare system**	
**Quote number**	**Quote**	**Participant**
2.01	“I mean, there's been a lot of trial and error to find out what's going to work and what doesn't work.”	Participant 3, Group 1
2.02	“They have to figure out, they have to do some trial and error, figure out what's going to work for each individual.”	Participant 3, Group 1
2.03	“I consider my treatments unsuccessful overall”	Participant 1, Group 1
2.04	“I was like, ‘What is this? What is going on?' I went to a doctor who diagnosed me with prostatitis, I got that first prostatitis diagnosis, he was treating me with some antibiotics, he tried some other different drugs to shrink the prostate, things like that…wasn't really making any headway and decided to switch doctors and go talk to someone else.”	Participant 2, Group 1
2.05	“I'm really struggling with a decision right now on whether I want to do that or not. I told my wife a couple of weeks ago, I said, ‘I don't mean this the way it sounds, but sometimes I wish I had bladder cancer so they could just go in and take it out and I wouldn't have a choice in it.' But, see, it's now coming down to me. Doctor says, ‘You either live with it like you're doing now and try to tweak things to make it better or you have the cystectomy and I will do it.”'	Participant 1, Group 1
2.06	And we've tried everything. So, I haven't been living well with it. We tried all the medications. The last thing we tried, we did get an implant. I've got an inter stem in my rear end that did not work. We tried it for 6 months. Adjusting it, trying to see if it would slow down the urge. And it did not work. So it's just turned off now.”	Participant 1, Group 1
2.07	“I've tried, like we've said, a number of different things, both prescription as well as non-prescription. One I mentioned earlier was about a tree leaf. The urologist suggested that, that didn't work. Nothing seems to work other than just living with it.”	Participant 4, Group 1
2.08	“I've got a good doctor that's been helping me and it's been helping me move through and fine ways to manage my symptoms.”	Participant 2, Group 1
**Theme 3**	**Emotional wellness**	
**Quote number**	**Quote**	**Participant**
2.09	“I was diagnosed with IC, officially, about 10 years ago because for several years before that I was constantly going to the bathroom, I didn't know why. I talked to my doctor about it and he tried a number of different things. I think I had four or five different medications to try to get the urination under control.”	Participant 4, Group 1
3.01	“…But it's psychologically frustrating to just know that something hurts but be told that you have a clean bill of health”	Participant 5, Group 2
3.02	“It gets me worried which causes stress which kind of just keeps feeding itself and it's hard to shut it down and get yourself in a state where, it hurts so bad, you know you have to go and you're trying to tell yourself to calm down because you're just making this worse and it kind of feeds on itself.”	Participant 2, Group 1
3.03	“I think for me over the years is what it's done is it's... there's just a lot of internal noise and a lot of internal conflict that I probably don't know how to process. And so, I think it probably comes out from time to time in anger management issues and things of that nature.”	Participant 3, Group 2
3.04	“I've noticed there's a lot of bottled up frustration, or like I said, anger.”	Participant 3, Group 2
3.05	“I don't really know if I… I don't really think I have mental issues with this other than the anxiety and frustration and things like that, which I tend to roll over anyway.”	Participant 1, Group 2
3.06	“Wishing for something like cancer? Yes. People understand that. They get it if you say that you have cancer. And it's something where, “I wish it would either kill me or get better.” I prayed that to god plenty of times. Make a decision. Kill me or make a decision. Kill me or heal me. Do one of the two, please.”'	Participant 2, Group 1
3.07	“There have been times when I, yeah, I've just said things that I don't think I would have said if I would have been fighting the pain”	Participant 3, Group 2
3.08	In other words, when I'm having a lot of issues, a lot of pain and so forth, my hypothesis is, I'm so busy fighting that, that I keep my emotions in check. And it's actually, it's the inverses. It's when I actually have a really good weekend or whatever time period, a few days or a day or afternoon, that's when it all, the emotions, I think, they want to catch up. And that's when, at times, I've noticed that there's a lot of bottled up frustration, or like I said anger.	Participant 3, Group 2
3.09	“I think emotionality with anxiety makes it worse. It's like a sympathetic activation. So, that causes you to want to be in control of every situation because you know the person that's doing the situation, whether it's an airline pilot or someone you're riding with, you know, has no idea of the things you go through with this.”	Participant 1, Group 2
3.10	“You know, when you don't know anyone else that's going through it, you start to doubt yourself a little bit. You start to think that you're a little bit crazy. And so, getting to sit down with people and they start telling the story and it's like, “That happened to me. That happened to me. That happened to me.” That was one of the most helpful things, I think, for me.”	Participant 2, Group 1
3.11	“I had a very strong feeling of isolation, until I started meeting other people who were going through it”	Participant 2, Group 1
**Theme 4**	**Coping**	
**Quote number**	**Quote**	**Participant**
4.01	“I've worn adult diapers when I go on a flight, just in case we get into turbulence and I can't go to the restroom. I make sure that I'm prepared, from that angle. I haven't ever had to use them, but they did give me a safety feeling, that if I have to go I can go.”	Participant 1, Group 1
4.02	“Do you know what a trucker's friend is? It's the bottle that they use in hospitals that men urinate it. I carry those in my vehicle and I have some extra ones just in case. Because, you know, you have to go, you have to go.”	Participant 4, Group 1
4.03	“If we're going to the movie theater, I don't sit in the middle of the row in the movie theater. I sit right on the end of an aisle. I went out to eat with some friends and I make sure I sit on the end of the bench in the booth because I don't want somebody to push me in toward the inside because just being in that situation too, that can start raising my stress levels and then I may not be enjoying the conversation, I'm just thinking or in the past definitely, I would be thinking, “How long can I make it before I've have to get up and ask this person to move?” And so, things like that start going into your mind and you start planning these things out that you never did before.”	Participant 2, Group 1
4.04	“I thought, “Okay. I'm going to have to read up on this and do a lot of talking to different people to figure out what's going on.””	Participant 1, Group 1
4.05	“I ended up coming across a book called Headache in the Pelvis and that actually helped me more than anything. I think I had been dealing with this for a little over a year, maybe two at this point. And going with different doctors, trying different drugs. Just trying to make any kind of headway that I can. Trying different diet things. I would drastically change my diets, that didn't seem to be having really any effect on my symptoms.	Participant 2, Group 1
4.06	“Well, I've made it a point to tell everybody that I have it, simply because I want them to understand why I have to get up from the table during the middle of dinner three times to go to the bathroom. So, everybody's been very understanding on that issue.”	Participant 1, Group 1
4.07	“I actually, about five, 6 months ago, we took a vacation over to the Netherlands and I explained to the friends we were meeting over there that if we were going traveling around…I needed to know where the restrooms were in the trains, if we were driving, I'd just let them know, “Hey, if I ask to stop to use a restroom, it's not because I'm being difficult, it just has to happen.” It's letting the people in my life know, there are certain things that I need to know and have taken care of. It's a lot of planning…just taking precautions.”	Participant 5, Group 1
4.08	“The way I explain it to people is that, it's kind of like static on a radio for me. The more stressed out I am and the higher my symptoms are, it's like you're turning the volume up on that static. And it'll get turned up to the point where you can't really focus on anything else but what you're feeling at that point.”	Participant 2, Group 1
**Theme 5**	**Treatment needs**	
**Quote number**	**Quote**	**Participant**
5.01	Mine is mostly about frequency and not knowing how something I eat or drink is going to affect me or stress or when it's going to occur…so, it is about the frequency. It's really controlling that and managing that and dealing with that more than anything else.	Participant 4, Group 1
5.02	“I think just sleeping through the night would help. I know everybody's had to stay up all night and they just feel groggy the next day, but do that every day for over a year. You can't think coherently at all	Participant 3, Group 1
5.03	“Something (a treatment) that would slow my bladder down from producing urine. And I've tried all these different prescription and non-prescription drugs that have not worked and I think if there was something that would slow that production of urine down for me, it would help a lot and I would do it in a flash, it's horrible and inconvenient.”	Participant 4, Group 1
5.04	“With me, it depends on which (symptom) is acting up more…So, I don't know. Yeah, so it is going to depend on the day in terms of which one is going to be more important (factor to treat IC).”	Participant 3, Group 1
5.05	“I was going to say more research. It's not a sexy disease like cancer or AIDS or things that get a lot of research dollars. I don't even know what IC would get, because it just seems like it could be one of those, it affects such a small amount of the population, that allocating a lot of funds to research probably isn't high on the priority list of the folks who are in charge of that. But, that's what I would say, more research into it to help define the problem and hopefully find better treatments.”	Participant 2, Group 1
5.06	“But, maybe there needs to be more focus on what these studies, what I'm trying to say is that more studies would involve men so that we can find out more about what we can do to help men. Because men do have similar symptoms, but different symptoms than women do, what I've been reading about it.”	Participant 4, Group 1
5.07	“I think that's something that's important for doctors to recognize- know when you're out of your depth. Don't be afraid to send them somewhere else. And even if you are the IC doctor, don't be afraid to send them somewhere else to get other opinions too. This has been a long road for me and I've seen a lot of different people and I think I got a little bit of something from every person that I saw, so. Some more than others.	Participant 2, Group 1
5.08	“I could see where that group therapy would help you. I would want to start with the individual doctor, psychiatrist to see where I am on this thing. If it is something that's mental or is it more physical. And then, I might consider group therapy, if it was something that, it's minimal and it would help you to be with others to talk and learn.”	Participant 4, Group 1
5.09	I would say group (would prefer group therapy instead of individual therapy). That was one of the reasons, when I saw this (focus group) study, I jumped on it immediately. I wanted to hear other people's stories. Because sometimes I questioned, is mine as bad as I think it is? Is it bad enough for me to consider a cystectomy? And so, I thought, “Well, this will give me an idea of what other people are going through and what they've done or what they're doing.” So, I would think a group therapy would be much better for me than individual.”	Participant 1, Group 1

The impact of IC/BPS was characterized as severe with reduced ability to work (*quotation 1.06*), participate in usual social engagements *(quotation 1.07*) and impact on sexual intimacy (*quotation 1.08*) and familial relations (*quotation 1.09*). This was comparable with the impact reported by women.

#### Healthcare system

Men also experienced difficulties in navigating the healthcare system and getting appropriate care. Men similarly described diagnosis and treatment as a trial-and-error process *(quotation 2.01, 2.02*) with an overall perception that treatments received were not efficacious (*quotation 2.03*). Key difficulties in interactions with the healthcare system included getting a diagnosis and finding a physician with the relevant knowledge (*quotation 2.04*). However, while men appeared to be actively included in treatment decisions by their healthcare professionals (*quotation, 2.08*), women commonly experienced dismissal (“*you are dismissed and you're, “Well, it ain't that bad”*) (17).

Some men experienced inclusion in treatment decisions as burdensome due to uncertainty about the best course of action (*quotation 2.05)*. Men also appeared to feel more definitively that they had reached the end of their treatment options (*quotation 2.06, 2.07*) in comparison to women who felt uninformed and the need to advocate for themselves. All men interviewed bar one perceived their symptoms to have been taken seriously, despite difficulties in receiving a diagnosis.

#### Emotional wellness

Men, like women reported a sense of “going crazy” also sought out validation from others for their experience, to confirm that they were not “going crazy” *(quotation 3.10)* and to combat feelings of isolation that came with the condition *(quotation 3.11)*. Some men saw a connection between their emotional state and their symptoms (*quotation 3.02, 3.09*), however this was less universal, clear and commonly explored compared to women who frequently reflected on this relationship *[“I'm wondering if …this anxiety and depression and stuff of that nature, the emotional sides of things, has not got something to do with this (IC/BPS symptoms)”*] (17). For men, the discussion of emotional experiences focused on feelings of stress, worry, anxiety and anger (*quotation 3.01*) as opposed to feelings of sadness emphasized by women (“*I'm saddened that I have something that I don't know what causes it”)* (17). Men also commonly reported the experience of suppressing emotions, with some difficulty in expressing and processing emotions related to the symptom experience (*quotations 3.03–3.05*). One of the ten men interviewed described severe emotional reactions with suicidal thoughts (*quotation 3.06)*. Generally, across the sample of men, emotions tended to manifest involuntarily in short tempers (*quotation 3.07*) or were deliberately avoided through suppression (*quotation 3.08*) and the use of pragmatic measures such as planning (*quotation 3.09*). This finding contrasted with the emotional experience of women, which was often described as prolonged and overwhelming (“*my emotions just kind of overwhelm me*”) (17).

#### Coping

Men approached coping with an action-oriented framework, using carefully planned safety strategies to avoid embarrassment from symptoms and associated stress. Specific coping behaviors included practical safety measures such as carrying extra items, wearing diapers, and sitting near the aisle for an ease of exit (*quotation 4.01–4.03*). Men also reported doing extensive research to become experts in their own condition (*quotation 4.04*), further allowing them to exert more control by implementing strategies such as changing diets and trying new medications (*quotation 4.05*). While women also conducted research and identified practical strategies for coping, men's analysis of problems focused on the practical aspects (e.g., availability of rest stops to urinate). Women took into account more often and to an apparent higher degree, the emotional layers of these considerations (e.g., the inconvenience to others or embarrassment of needing additional rest stops) (“*I don't want to be a burden to them*”) (17).

Rather than withdraw from relationships due to lack of perceived support or understanding as reported by women (17), men felt comfortable asserting their needs as arising from the condition with friends and family. These included needing to stop to use the bathroom (*quotations 4.06, 4.07*) and being at a reduced capacity for activity engagement due to pain (*quotation 4.08*).

#### Treatment needs

Men focused on the need for more practical and medical developments to improve symptom experience. Frequency was a key symptom they identified as wanting help reducing, particularly regarding its role in sleep disruption (*quotation 5.01–5.03*). It was also acknowledged that symptoms fluctuated, making it difficult to identify one key symptom treatment that needed prioritizing (*quotation 5.04*). There was an overall desire for more research and awareness for IC/BPS (*quotation 5.05*) particularly in relation to IC/BPS within men, with a sense that IC/BPS treatment tailored to IC/BPS in men specifically could be useful (*quotation 5.06*). Men expressed a need for healthcare professional transparency where professionals lacked sufficient knowledge and experience, so that they may be referred on to a more informed practitioner who could provide them with more effective care options (*quotation 5.07*). Unlike women who readily identified the importance of mental health support, particularly integrated with the experience of IC/BPS [“*how can I find someone that I can sit down and talk about both (anxiety and IC/BPS) …I think it'd be helpful to have some type of different help…having a mental help might be good*”] (17) men did not appear to prioritize the need for psychological support and only considered it's utility when prompted by the interviewer. In speculating on the role of mental health support, men tended to focus on the practical support benefits and normalizing effects that group therapy could have (*quotation 5.09*), while one participant specified wanting to see an individual practitioner first (*quotation 5.08)*.

## Discussion

In this investigation, gender differences emerged in both assessments of symptom profiles of men and women with IC/BPS and in systematic patient interviews discussing patient experiences of symptoms and their impact. In examining qualitative themes of men's IC/BPS experiences, key contrasts emerged in comparison to women (17) involving emotional engagement as it relates to IC/BPS, coping strategies used, communication with others regarding symptoms, and in experiences navigating the healthcare system. These observed gender differences appear fundamentally rooted in the experience of and response to pain, both internally and externally by the social environment.

In this sample, women reported different physiological experiences of pain. While both genders experienced moderate levels of urologic symptoms, women reported significantly greater problems due to urologic symptoms, pain intensity levels, and degree of widespread pain throughout the body than men. Both groups had similar levels of impairment due to symptoms. In qualitative interviews, while pain seemed central to women's experiences, men expressed more concern with urinary symptoms and their impact.

Experiences also differed in internal reactions to symptoms between genders. On assessments, both men and women self-reported elevated levels of depression and anxiety. However, qualitatively, although men acknowledged some stress and frustration in response to IC/BPS symptoms, emotional distress was not as central to their illness experience as it appears for women (17). Men used less varied adjectives than women to describe their emotional experience, with more undifferentiated ways of describing their emotional experience (“*there's just a lot of internal noise and a lot of internal conflict that I probably don't know how to process”*) with some appearing not to acknowledge the emotional experience they described: “*I don't really think I have mental issues with this other than the anxiety and frustration and things like that, which I tend to roll over anyway.”* In varying ways, men described processes of bottling up frustration, anger and stress without apparent clarity on that process. Although a small proportion of men interviewed acknowledged the exacerbating role emotions may have in the experience of symptoms, there was less exploration and description about the reciprocal relationship between symptoms than in interviews with women.

These findings could suggest alexithymic traits amongst men with IC/BPS. Alexithymia is a reduced ability to identify and describe feelings and is more prevalent amongst some conditions including interstitial cystitis (27, 28). Rather than the “absence” of emotion, results of interviews and qualitative coding suggested that men experienced a significant emotional impact due to IC/BPS but had a reduced vocabulary or tendency to express affective experience. For example, men noted the importance of having others to speak with but did not use the word “loneliness.” Participants discussed at length the excessive cognitive and planning burden due to IC/BPS symptoms, but the word most commonly used to describe emotional experience of this was “frustrating.” This finding is in line with the “normative male alexithymia” hypothesis (29) which theorizes a socialized pattern of emotional restriction in men that aligns with traditional gender roles, with men having particular difficulty verbalizing emotions expressing vulnerability or attachment (29, 30).

In comparison, women qualitatively reported a greater range of emotions including episodic severe depression, anxiety, and feelings of guilt following IC/BPS symptoms, with a clear perception of the bidirectional relationship between symptoms and affective distress (17). Yet it is important to consider that the present findings could be influenced by men's potential discomfort with emotional disclosure in the presence of others due to masculinity norms (10).

The absence of guilt in men's experience of IC/BPS may be related to the different coping strategies used between genders. Men felt more able to assert their needs to family and friends than women. Further, men did not withdraw socially as much as women, who reported isolating and feeling burdensome to others (17). Men employed more proactive, task-oriented coping such as strategically planning activities around toileting needs, trying new medications, and carrying extra items in case of an accident. This style of coping is generally found to be adaptive in long term health conditions, empowering patients while preventing emotional overwhelm (31, 32). It is, however, necessary to make the distinction between adaptive coping and emotional suppression as emotional suppression/avoidance is negatively associated with physical and psychological outcomes in chronic illness and can exacerbate pain (33). The lack of focus on the emotional impact of IC/BPS could suggest that men actively engage in emotional suppression. Previously it has been found that task-oriented coping is positively associated with emotional suppression (33).

The differences in coping styles between genders was also reflected in the discussion of perceived treatment needs. Men did not actively consider the need for social support or mental health support, which was strongly emphasized by women (17). Instead, men wanted more practical and medical developments, with a preference of healthcare professional transparency regarding familiarity and experience with IC/BPS. This distinction between men and women could relate to differing healthcare system experiences. Although both men and women reported a difficult path to diagnosis, with trial-and-error treatment processes, a key distinction in this journey was how each gender felt supported by healthcare professionals. Men reported feeling believed, validated, and supported by healthcare professionals whilst women felt disbelieved and dismissed. This was a finding potentially quantitatively reflected in the differences in time between symptom onset and diagnosis, which was much longer for women than men. Men reported experiencing shared decision making, a key component of patient-centered care (34) that was not reported by women. Negative interactions with providers may also relate back to different treatment needs between genders, with women reporting an increased need for emotional support. Studies show that healthcare professionals find it harder to adequately respond to distress in consultations (35), which could partially account for the gender differences in navigating patient-provider interactions.

Differences between genders in satisfaction with healthcare professionals' communication and support has been found in other patient populations. Our findings are consistent with the wider literature, with men rating communication with physicians more positively than women (35). In a recent survey of women's experiences of accessing healthcare for urogynaecological issues, 84% of women reported experiences of dismissal in the healthcare system (36). Stigma from healthcare professionals is commonly perceived as problematic by patients in the treatment of chronic pain, particularly when female (37). This problem is found to be compounded by negative stereotypes about women (e.g., that distress signifies that pain is psychological). However, it is also important to consider that the disparity in satisfaction between men and women could be also due to differences in aspects of communication that may be valued more or less based partially on gender. For instance, research suggests that female patients ask more questions, provide more comprehensive histories and express emotions more, while men communicate more forcefully and are more task- oriented (38). Our findings in the context of the wider literature point to the importance of a focus on individualized patient communication with shared-decision making at the heart of consultations.

The pain symptom elevations observed in women in this cohort parallels previous studies (39). It is important to consider treatment implications of study findings. National AUA guidelines urge both multidisciplinary management and the incorporation of psychosocial stress management for IC/BPS (40). It is possible, based on study findings, that such interventions may be received differently by gender. Although men may benefit from psychological services to support their treatment, additional care may be needed in explaining the rationale for these services to prompt referral and subsequent engagement. Specifically, men may need further explanation of the relationship between stress and urologic symptoms, and potential for behavioral strategies to assist with sleep hygiene and bladder training to address urinary symptoms and associated sleep disruption—which appear more bothersome to men than women. Other aspects of psychosocial interventions for IC/BPS may benefit from tailoring according to presentation as potentially impacted by gender. Men felt competent in handling the practicalities of their condition, however they potentially neglect the important emotional impact of IC/BPS. Research suggests that unprocessed emotion is highly related to the experience of pain (28, 33). Some studies suggest that men would benefit from a wider arrange of coping strategies, including emotion-focused coping, and that this could help to improve functional outcomes (7, 41, 42). Therefore, psychosocial interventions aimed at facilitating the processing of emotions may focus on identifying and acknowledging distress in men and expanding emotion-focused coping strategies where possible. For women, treatment may focus more on processing, containing, and facilitating adaptive problem-solving coping responses to pain and distress to expand coping strategies.

Psychological interventions for IC/BPS are a new and under-addressed area of study. This study provided an in-depth assessment of how IC/BPS affects the lives of both men and women. The detailed interviews gave additional insight into the nuances of coping and managing urologic pain and urinary symptoms, and also exposed important differences in experiences that may inform intervention development and areas to improve patient interactions. Regarding study limitations, due to the qualitative nature of the investigation, thematic saturation in focus group discussions drove this study's sample size. Thematic code and meaning saturation can be obtained in five or more focus groups (43). When exploring issues by demographic strata, it is recommended to conduct two focus groups per stratum to capture nuances of conceptual codes (43). Thus, this study achieved an appropriate sample to achieve its qualitative aims. However, interpretation of statistical comparisons is limited by sample size. The overall study sample had similar demographics (white, middle aged). Therefore, the results of the present study may not be representative of the experience of the wider population of men and women with IC/BPS. In assessing emotional wellness, under-reporting of depression has been found amongst men and older adults in other contexts both on self-report measures and with direct inquiry (44). Consequently, the results of this study may have been affected by men not feeling comfortable in the context of a group to discuss their depressive and emotional symptoms at length. This may itself inform intervention efforts, as men may be more comfortable with individual intervention. Further, men may benefit from interventions requiring less emotional identification, such as systemized bladder training, a first line behavioral treatment focused on modification of voiding *via* monitoring and delaying voids systematically. This training, which requires behavioral and cognitive, but not emotional responses to symptoms, may be particularly well-suited to men's preferred intervention strategies (45, 46).

Biopsychosocial approaches to pain conditions such as IC/BPS identify the role of complex social and psychological processes in a person's experience of an illness. This study identifies two potential areas of influence that differ by gender for biopsychosocial conceptualizations of IC/BPS: interactions with the healthcare system and emotional processing of symptom experiences. While this investigation marks distinctions in the experiences of IC/BPS between genders, it cannot demonstrate that gender is the driving factor of all differences found such as the varied levels of symptom severity reported between men and women in this study. Higher levels of polysomatic complaints and pain can also influence healthcare interactions and treatment satisfaction (47). Research suggests that people earlier in their treatment, or more recently diagnosed (which in this sample would be men) are more vulnerable to depression and emotional distress (15). This was contrary to our findings, which indicates the need for further research exploring the relationship between gender, outcomes and healthcare experiences including diagnostic journeys.

These results suggest that gender health inequality may exist in the healthcare experiences of IC/BPS. Current practices seem to provide satisfactory patient-centered care for men but not for women. Provider training in the effect of disbelief and dismissal may benefit clinical practice. Furthermore, women experiencing distress due to their IC/BPS may require additional validation, empathy, and support in order to facilitate patient-centered care and meet women's perceived treatment needs from providers.

## Data availability statement

The raw data supporting the conclusions of this article will be made available by the authors, without undue reservation.

## Ethics statement

The studies involving human participants were reviewed and approved by Institutional Review Board, Vanderbilt University Medical Center. The patients/participants provided their written informed consent to participate in this study.

## Author contributions

LM, MF, WR, and RD were involved in the conception and design of the study. LM, MF, KB, and DS implemented the study. MF, KB, DS, SW, SS, and LM were involved in the quantitative and qualitative analysis of data. All authors were involved with writing and reviewing the manuscript and gave final approval for submission.

## Funding

This project was conducted with support from the Vanderbilt Patient Centered Outcomes Research Education and Training Initiative and the Agency for Healthcare Research and Quality (AHRQ 6K12HS022990-05), National Institute of Health (National Institute of Diabetes and Digestive and Kidney Diseases) Award DK118118, and by Vanderbilt University Medical Center CTSA Award Nos. UL1TR000445 and UL1TR002243 from the National Center for Advancing Translational Sciences.

## Conflict of interest

The authors declare that the research was conducted in the absence of any commercial or financial relationships that could be construed as a potential conflict of interest.

## Publisher's note

All claims expressed in this article are solely those of the authors and do not necessarily represent those of their affiliated organizations, or those of the publisher, the editors and the reviewers. Any product that may be evaluated in this article, or claim that may be made by its manufacturer, is not guaranteed or endorsed by the publisher.

## Author disclaimer

Its contents are solely the responsibility of the authors and do not necessarily represent official views of the National Center for Advancing Translational Sciences or the National Institutes of Health.
